# Ready for Change!

**DOI:** 10.5334/jbr-btr.1055

**Published:** 2016-02-11

**Authors:** Alain Nchimi

**Affiliations:** 1 University of Liège, Belgium, BE

“If we want things to stay as they are, things will have to change.”

Giuseppe Tomasi di Lampedusa, The Leopard

*“Change is the law of life and those who look only to the past or present are certain to miss the future*.”

John F. Kennedy

The Journal of the Belgian Society of Radiology (JBSR) has been subject to profound modifications within a short time frame during the past year. First, the journal is now operated fully online for submission, review and publication. This means the last printed regular issue of the journal was the JBR-BTR 98/1 issue in 2015 (Figure 1). This has been disheartening for those of us who like true books, paperwork and collections. However, it is part of the price to pay for expediting the review and publication processes, although, its implementation has led to a considerable backlog that paradoxically slowed it down [1]. The JBSR concedes no exception to other scientific journals, with its peer-reviewers pool being its most important resource. By the end of 2015, we are grateful our peer-reviewers reviewed nothing less than 210 manuscripts and considerably reduced the burden of manuscripts awaiting an editorial decision. We hope our peer-reviewers continue to remain committed and provide the now–expected–growth of the JBSR. We are happy to be able to track and manage every manuscript in nearly real-time, from submission to archive. In addition, the online operation platform offers the editorial staff a powerful tool to manage and quantify the workload of the peer reviewers so that they can maintain regular activities for the journal, without excessive solicitations.

**Figure 1:**
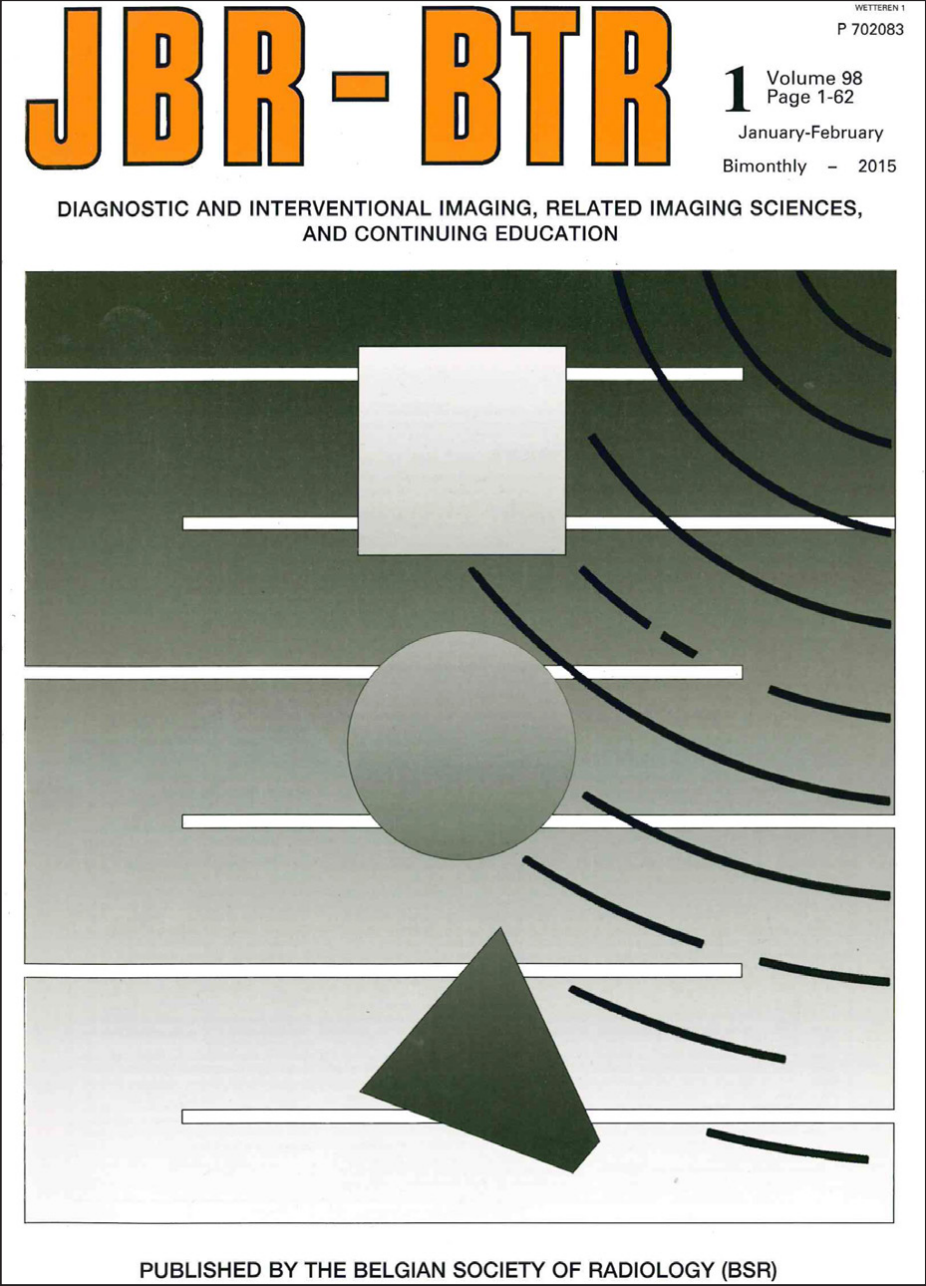
Cover of the JBR-BTR 98/1, the last printed issue of the journal.

Second, after successfully leading the move of what we should call *his* journal into the fully online era, Prof. Jacques Pringot has called time to his career as editor-in-chief of the JBSR and kindly accepted to share his experience serving as emeritus editor-in-chief. Prof. Jacques Pringot has been a role model for several generations of radiologists and a stalwart to the journal since the beginning of his academic career at the Cliniques Universitaires Saint-Luc and since he became the journal’s editor-in-chief in 1974 (Figure 2). He has supervised the publication of 240 journal issues and more than 3,800 articles. His name is, and will remain, associated with the journal for decades. Taking over after such an impressive achievement may frighten a normal person, and so we are. However, we are aware that a collective approach will transcend most difficulties, and we are full of hope that the difficulty of missing our preferred editor-in-chief will be overcome, when we all (stakeholders, editors, publisher, authors and reviewers) act as a team serving our readership. As from the appointment of its new editor-in-chief, the editorial staff has been fortunate to benefit from the services of Mrs. Katrin Lorent, the new Assistant-Editor, and Dr Piet Vanhoenacker who has been the instrumental Managing-Editor of the JBSR for the last two years, being pivotal in the selection of the publisher and kick-off of the new electronic platform. The choice for the current publisher was made for its goals of sustainability in publishing. Ubiquity Press is a young company that started up as spin-off of the University College London, and promises to “operate a highly cost-efficient model that makes quality open access publishing affordable for everyone” [2]. Of course we will seek to gradually expand our staff in the next months, in line with the needs that will be progressively unveiled.

**Figure 2:**
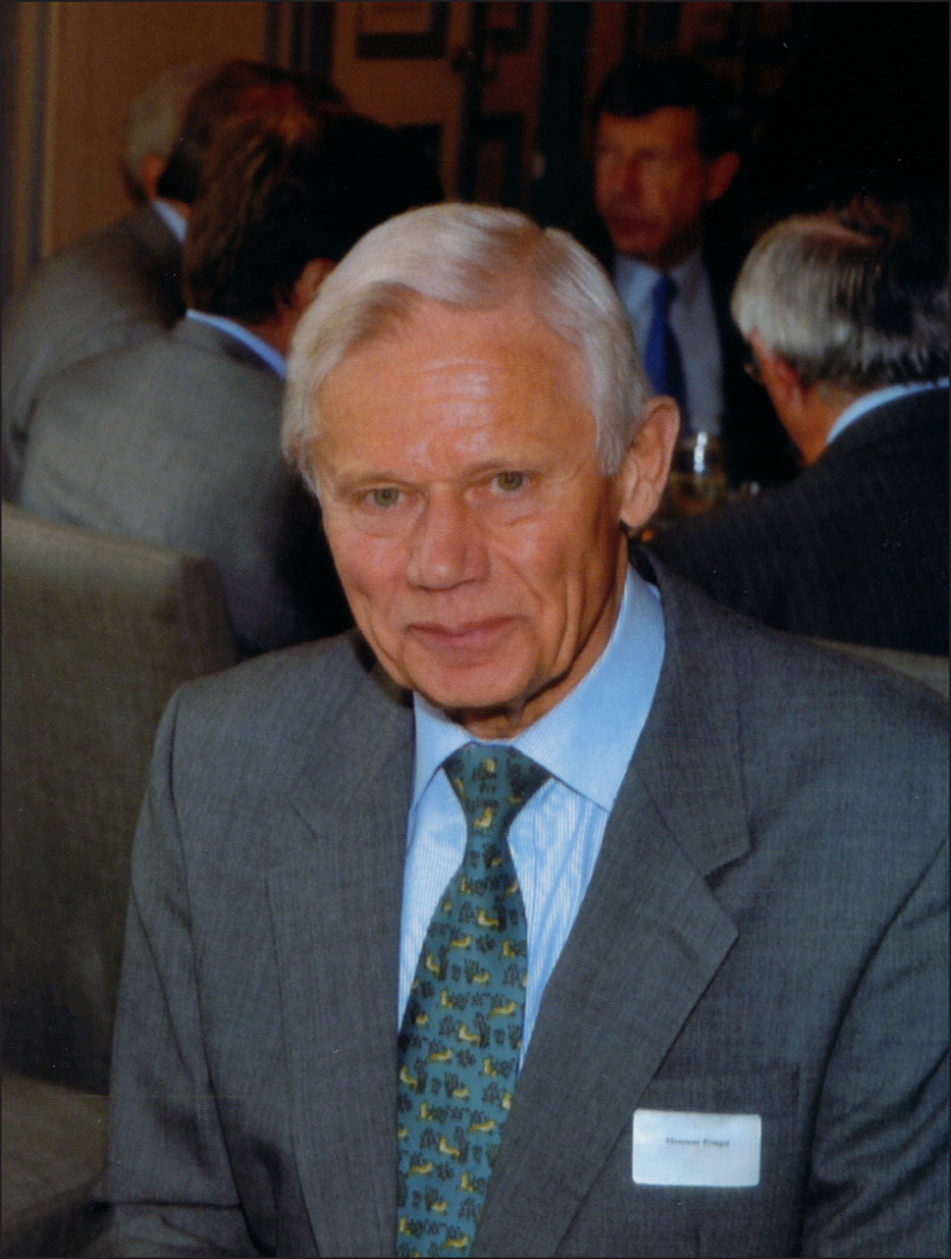
Jacques Pringot, Emeritus editor-in-chief of the JBSR.

Third, the board of the BSR has decided to change the journal’s name from JBR-BTR (Journal Belge de Radiologie-Belgisch Tijdschrift voor Radiologie) to become JBSR. One may consider the aforementioned changes are way too much and that the upcoming year should be less hectic, but thinking so is probably wrong in the post-internet era, characterized by rapid transnational communications. Amongst many other changes to come, the metrics and article automatic indexation of the JBR-BTR will be allocated to the JBSR after all due verifications. Meanwhile, we will define and implement the article types we want to encourage and how we want these to be disseminated and debated in and outside our community. Of course we will commit ourselves to education and scientific promotion that are among the goals of the BSR. By reciprocity, we expect from the BSR a greater contribution to the journal’s quality and scientific content through its scientific subsections, starting by nominating new editorial board members, ready to take all ongoing changes. Actually, if we do not take the opportunity to change things, we just surrender to changes imposed by others. It is well established that for sustainable progress, change is just a minimal requirement that prepares others to change. In this sense, the change of our journals’ name means more than a simple cosmetic facelift and epitomizes the difficult choices we are facing when implementing changes.

Under its previous denomination, the JBSR already published articles in English, the indisputable standard of communication for biomedical research. By removing references to our main national languages in its official journal’s title, the board of the BSR made a strong statement towards opening its interaction and broadening its base. However, in this political realm of anxiety to lose traditions caused by too much openness, the board of the BSR has also decided that the name of our national society will remain in the journal’s title. This does not only affirm our need to be recognized as such by all, but also subliminally tells who we are: Whatever the changes we are facing, we remain strongly attached to Belgian traditions, among which, a political sense of consensus where people often wisely decide to arrange the goat and the cabbage. Whatever its name, the need to define our journal as fully open to the world is just an example of the many decisions and changes we have to implement this year and in the years to come. Isn’t the change continuous?

## Competing Interests

The author declares that they have no competing interests.
